# Optimization and Characterization of the Synthetic Secondary Chromosome synVicII in *Escherichia coli*

**DOI:** 10.3389/fbioe.2016.00096

**Published:** 2016-12-23

**Authors:** Sonja J. Messerschmidt, Daniel Schindler, Celine M. Zumkeller, Franziska S. Kemter, Nadine Schallopp, Torsten Waldminghaus

**Affiliations:** ^1^LOEWE Center for Synthetic Microbiology, SYNMIKRO, Philipps-Universität Marburg, Marburg, Germany

**Keywords:** genetic engineering, bacteria, synthetic chromosomes, synthetic genomes, *Vibrio cholerae*, chromosome biology, replicon integrity, directed evolution

## Abstract

Learning by building is one of the core ideas of synthetic biology research. Consequently, building synthetic chromosomes is the way to fully understand chromosome characteristics. The last years have seen exciting synthetic chromosome studies. We had previously introduced the synthetic secondary chromosome synVicII in *Escherichia coli*. It is based on the replication mechanism of the secondary chromosome in *Vibrio cholerae*. Here, we present a detailed analysis of its genetic characteristics and a selection approach to optimize replicon stability. We probe the origin diversity of secondary chromosomes from *Vibrionaceae* by construction of several new respective replicons. Finally, we present a synVicII version 2.0 with several innovations including its full compatibility with the popular modular cloning (MoClo) assembly system.

## Introduction

New DNA-assembly methods have been developed in recent years, and costs of DNA synthesis are constantly decreasing (Kosuri and Church, 2014; Chao et al., 2015). These two factors are the main driving force for an increasing number of synthetic chromosome projects (Gibson et al., 2008; Lee et al., 2013; Annaluru et al., 2014; Schindler and Waldminghaus, 2015). This development was started by Venter and coworkers who constructed a whole *Mycoplasma genitalium* chromosome with a size of 583 kb from scratch (Gibson et al., 2008). Two years later, a synthetic chromosome was introduced into bacterial cells replacing the natural chromosome (Gibson et al., 2010). The two synthetic chromosomes had in common that they were basically copies of natural genome sequences. The next thing to do would consequently be using the new methodologies to engineer on a chromosome-wide scale. The stepwise replacement of chromosome III in *Saccharomyces cerevisiae* with a designed synthetic chromosome synIII was a step in this direction (Annaluru et al., 2014). In addition, genome-wide recoding of codons is now possible (Lajoie et al., 2013; Ostrov et al., 2016). However, eukaryotic and prokaryotic chromosomes are fundamentally different (Kuzminov, 2014). The same is true for their gene organization and expression mechanisms leading to the important question of how the genome as operating system fits to a specific chassis (Danchin, 2012). Notably, a *Mycoplasma* strain can be changed into another by transplantation of a chromosome showing that the genome as operating system can run on different chassis (Lartigue et al., 2007). Interestingly, the efficiency of transplantation decreases with increasing evolutionary distance between chromosome donor and recipient (Labroussaa et al., 2016). Such chromosome transplantation is fundamentally different from other hosts of synthetic chromosomes where the extra DNA is maintained within the cells, but the encoded information is not translated into function. One example is a complete *Synechocystis* genome within *Bacillus subtilis* (Itaya et al., 2005). In addition, yeast is now used frequently to harbor bacterial chromosomes to facilitate their modification using the extensive genetic toolbox available for *S. cerevisiae* (Benders et al., 2010; Karas et al., 2013, 2014). An alternative to changing the primary genome of an organism is the addition of extra replicons. In bacteria, the genetic content is generally stored on a single chromosome replicated from a single replication origin. A secondary copy of this replication origin as driver of an extra replicon has been shown to cause several problems probably due to competition with the native replication origin (Lobner-Olesen, 1999; Skarstad and Lobner-Olesen, 2003). One interesting alternative is the replication origin of the secondary chromosome of *Vibrio cholerae*. This origin has been shown to replicate in *Escherichia coli* and was used in several respective genome engineering projects (Egan and Waldor, 2003; Liang et al., 2013; Messerschmidt et al., 2015; Milbredt et al., 2016; Zhou et al., 2016).

*Vibrio cholerae* is a model system for multi-chromosome bacteria. Its primary chromosome (ChrI) has a size of 2.96 Mbp and the secondary chromosome (ChrII) a size of 1.07 Mbp (Heidelberg et al., 2000). While ChrI is replicated from the DnaA-controlled replication origin I (*oriI*), similar to *E. coli*, chrII is replicated from the RctB-controlled origin II (*oriII*) (Egan and Waldor, 2003; Duigou et al., 2006). Both chromosomes encode their own segregation systems (*parAB1* and *parAB2*) (Yamaichi et al., 2007b). The core *oriII* region is flanked by the *parAB2* and the *rctB* gene. ParB2 seems not only to participate in segregation but also in the regulation of DNA replication of ChrII (Yamaichi et al., 2011; Venkova-Canova et al., 2013). It binds specifically to parS2 sites occurring throughout chromosome II (Yamaichi et al., 2007a; Ramachandran et al., 2014). ParA2 binds DNA to form a left-handed helical filament (Hui et al., 2010). Formation of such ParA–DNA collaborative filaments is essential for DNA movement during cell cycle progression, but the underlying molecular mechanism remains to be uncovered (Ghosal and Lowe, 2015). The regulation of the replication timing in this two-chromosome system has been extensively studied over the last years (Egan et al., 2004; Rasmussen et al., 2007). It was shown that ChrI initiates DNA replication first followed by initiation at *oriII* after about two-thirds of the primary chromosome is replicated (Rasmussen et al., 2007; Stokke et al., 2011; Val et al., 2016).

On the basis of *oriII* from *V. cholerae* we previously constructed a prototype of the synthetic secondary chromosome synVicII in *E. coli* (Messerschmidt et al., 2015). Here, we present a thorough characterization and introduce several innovations leading to a new version of synVicII to satisfy the need for well understood and easy-to-use replication systems for bioengineering and synthetic biology applications.

## Results and Discussion

### Genetic Integrity of synVicII

**Figure 1:**
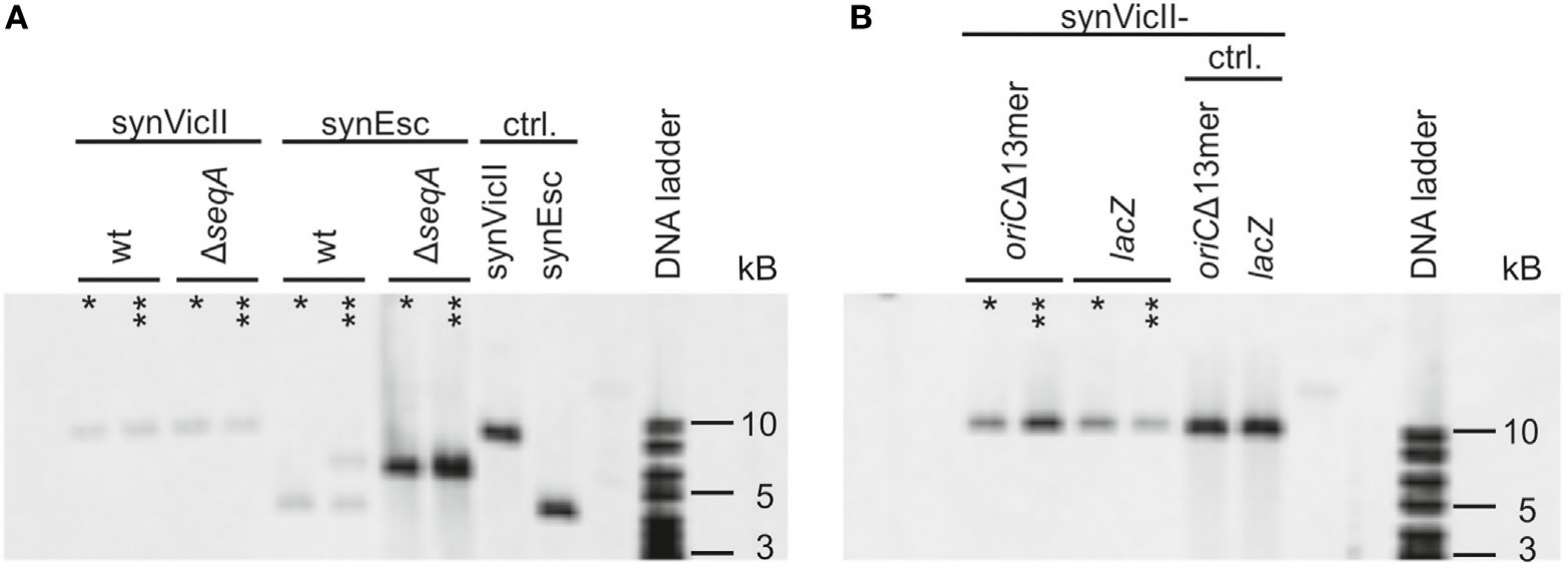
**synVicII does not integrate into the *Escherichia coli* chromosome**. **(A)** *E. coli* wild-type strains carrying either synVicII-1.3 (SMS18) or synEsc-1.31 (SMS65) and Δ*seqA* carrying synVicII-1.3 (SMS66) or synEsc-1.31 (SMS67) were grown exponentially over 3 days in LB medium at 37°C. Genomic DNA was extracted after 1 (one asterisk) or 3 days (two asterisks) and blotted as described in section “Materials and Methods” after digestion with *Nco*I. 1 µg of DNA was used for strains SMS18, SMS65, and SMS66; 500 ng from SMS 67 and 25 ng of synVicII synEsc. *Nco*I digestion linearizes synVicII and yields a 9,134 bp fragment and 4,378 bp for synEsc. **(B)** Sequences with homology to the *E. coli* chromosome were inserted into synVicII, either an inactive *oriC* (synVicII-1.301) or a part of *lacZ* (synVicII-1.302). Respective strains SMS72 and SMS74 were analyzed as above. Non-integrated linearized fragments are 9,891 bp fragment for synVicII-1.301 and a 10,199 bp fragment for synVicII-1.302 as seen for the control with purified replicons.

Genetic circuits for biotechnological applications might be integrated into the primary chromosome of a production strain or alternatively be placed on a secondary synthetic chromosome or plasmid. However, full control of the genetic setup is mandatory. Integration of a secondary replicon into another replicon may, for example, destroy its genetic context and attributes (Haldimann and Wanner, 2001). Notably, the use of an additional copy of the primary chromosome origin to drive secondary chromosome replication is known to result in frequent integration into the primary chromosome (Lobner-Olesen, 1999; Skarstad and Lobner-Olesen, 2003). To test if the synthetic secondary chromosome synVicII is also prone to integration into the primary *E. coli* chromosome, we measured the degree of integration after an extended cultivation of respective cultures for 1 or 3 days by Southern blot analysis (Figure 1). *E. coli* strain SMS65, carrying the *E. coli oriC*-based replicon synEsc was used as control. Total DNA from respective strains was digested with *Nco*I leading to linearization of non-integrated replicons. A potential integration would lead to a band shift of the detected DNA fragment. Such a shift was seen for a portion of cells carrying synEsc, while the synVicII DNA fragment was unchanged even after 3 days of continuous cultivation (Figure 1A).

The conditions of cell growth tested here might not fully reflect the diversity of conditions that a production strain might face. To simulate more challenging conditions regarding genetic integrity, we transferred synVicII to a strain with a DNA replication defect caused by a deletion of the SeqA protein (Lu et al., 1994; Waldminghaus and Skarstad, 2009). This strain background has been shown to increase the frequency of replicon integration into the primary chromosome (Skarstad and Lobner-Olesen, 2003). In fact, the control replicon synEsc was integrated throughout the population after only 1 day of cultivation (Figure 1A). In contrast, synVicII remained a separate replicon even after 3 days of continuous cultivation (Figure 1A).

A notable difference between the two replicons compared here is that synEsc contains sequences with homology (*oriC*) to the *E. coli* primary chromosome, while synVicII shares no homology with the *E. coli* chromosome. This raises the question if the different integration behavior of the replicons is caused by their ability to function in a homologous recombination reaction. To test if the synVicII-integration frequency is dependent on homologous sequences on the replicon, we inserted two different genetic regions that also occur on the *E. coli* chromosome. First, a synVicII version was constructed carrying 1,065 bp of *lacZ* (synVicII-1.302). Second, a copy of *oriC* was inserted similar to synEsc but made inactive by deletion of 44 bp in the initiation region (synVicII-1.301). Although these two versions of synVicII carried sequences with homology to the *E. coli* chromosome and thus being a potential target for homologous recombination, the replicons did not integrate but remained as separate entity (Figure 1B). Integration of *oriC*-based replicons into the primary chromosome was explained by competition of such replicons with the native *oriC* for initiation factors (Lobner-Olesen, 1999; Skarstad and Lobner-Olesen, 2003). Such competition seems not to occur for replicons as synVicII, based on *oriII* from *V. cholerae*. Notably, an *E. coli* system with *oriC* on the primary chromosome and *oriII* on a secondary chromosome mimics the genetic setup in *V. cholerae* where the two chromosomes are kept separate although exceptions have been observed (Johnson et al., 2015).

### Selection of Stabilized Versions of synVicII

**Figure 2:**
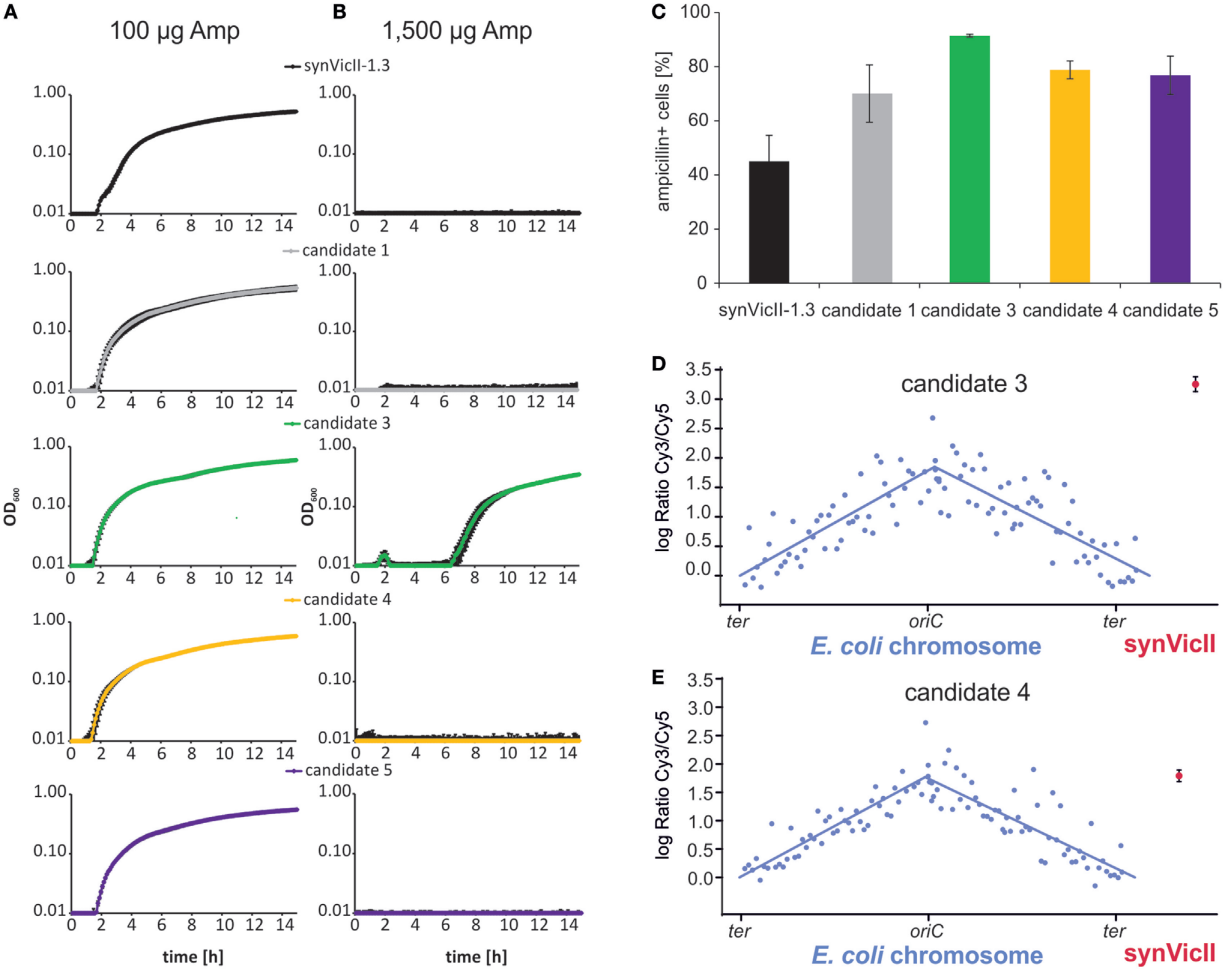
**Selection of stabilized versions of synVicII-1.3**. **(A,B)** Growth test to distinguish low-copy and high-copy versions of evolved synVicII versions. Candidate strains were grown in LB medium with low and high ampicillin concentrations as indicated in a 96-well plate at 37°C. **(C)** Stability of selected candidate versions of synVicII in comparison to synVicII-1.3. Given numbers are mean values of ampicillin-resistant cells after 6 h of cultivation without antibiotic selection from three biological replicates (Messerschmidt et al., 2015). **(D,E)** Comparative genomic hybridization of *Escherichia coli* strain SMS81 harboring synVicII-candidate 3 and SMS79 harboring synVicII-candidate 4, respectively. DNA of exponentially grown cells was hybridized against DNA of a hybridization control (see Materials and Methods for details). Respective logarithmic values of ratios are plotted against their genomic position. Blue dots represent the 104 probes for the *E. coli* chromosome, blue lines the linear curve fitted to chromosome arms. The red dot represents the mean of the three probes of synVicII with the corresponding standard deviation.

Different applications of a synthetic secondary chromosome might require different characteristics. For example, if the replicon is used to analyze the stabilizing effect of different genetic elements, it would be important to use a replicon, which is lost from a cell population over time under non-selective conditions. On the other hand, a replicon and its genetic content would need to be stably transmitted from cell to cell if it is used in biotechnological processes. Notably, the prototype of synVicII showed a certain degree of instability (Messerschmidt et al., 2015). The setup for a selection system to generate stabilized versions of synVicII is shown in Figure S1 in Supplementary Material. The basic idea is that *E. coli* cells carrying synVicII are cultivated without antibiotic selection. After some generations, a proportion of the population will have lost the replicon and others will still carry a synVicII copy. Versions of synVicII with a stabilizing mutation belong to the later ones and are selected by transfer to growth medium with antibiotic selection. Replication characteristics of secondary replicons can change due to mutations on the primary chromosome (Lopilato et al., 1986; Ederth et al., 2002). Since we were interested in mutations of synVicII itself, the replicons of individual clones were isolated and retransformed into a “clean” genetic background. Stabilizing mutations might target different mechanisms of replicon maintenance. One possibility is mutations leading to an increased copy number. A higher replicon copy number leads to increased stability because just by chance it is more likely for each daughter cell to get at least one replicon copy. In fact, amino acid changes in replication initiator proteins are frequently found to increase the replicon copy number (Fang et al., 1993; Wadood et al., 1997). This is also true for replicons based on *oriII* of *V. cholerae* similar to synVicII (Jha et al., 2012; Koch et al., 2012). Such copy-up mutations are not desirable for synVicII because one of its main features is its low-copy number comparable to the primary chromosome (Messerschmidt et al., 2015). We applied a simple screen for copy-up mutations by growing candidate clones in medium with different concentrations of ampicillin (Uhlin and Nordstrom, 1975; Carleton et al., 1984). The respective logic is that a higher copy number of the replicon correlates with a higher copy number of the β-lactamase gene and consequently its higher expression. Cells carrying a replicon with a higher copy number should therefore tolerate higher amounts of the β-lactam antibiotic ampicillin. Measuring the growth of cells with synVicII or one of four different evolved versions showed very similar growth in the standard ampicillin concentration of 100 µg/ml (Figure 2A). In contrast, only one strain grew at an elevated ampicillin concentration of 1,500 µg/ml, suggesting that this strain carries a copy-up mutation (Figure 2B). Increased stability of the candidates compared to the original synVicII was measured by the number of colony-forming units after 6 h of exponential growth without selection pressure (Figure 2C). To verify that our reasoning of the ampicillin-growth test was correct and to further characterize the evolved synVicII versions, we performed copy number measurements by comparative genomic hybridization on a custom-made microarray (Messerschmidt et al., 2015). Probes on the array match the *E. coli* chromosome as well as synVicII. DNAs from exponentially growing strains carrying the potentially copy-up mutation (candidate 3) or a non-copy-up version (candidate 4) were hybridized against the hybridization control of non-replicating *E. coli* strain FSK18 as described previously (Messerschmidt et al., 2015). Respective fluorescence ratios were plotted relative to the chromosomal position (Figures 2D,E). The relative abundance of chromosomal loci diminishes exponentially with increasing distance from the origin for exponentially growing populations as seen for the primary chromosome [Figures 2D,E; Sueoka and Yoshikawa (1965)]. Fitted curves were used to calculate average *oriC*/*ter* ratios, which were very similar for the two strains and within biological replicates (Table S5 and Figure S2 in Supplementary Material). In contrast, the copy number of the predicted copy-up version of synVicII was 9.5 relative to the terminus of the primary chromosome—almost three times higher than *oriC*. The predicted non-copy-up version of synVicII had a copy number of 3.5 very similar to the *oriC* copy number. We conclude that the selection approach introduced here is able to produce both, copy-up and non-copy-up versions of synVicII that are stabilized. Notably, the setup should in principle be suitable for any other secondary replicon optimization.

We hypothesized that the copy-up phenotype of candidate 3 was caused by a change in the amino acid sequence of the initiator protein RctB as found previously (Koch et al., 2012). By sequencing, we indeed found a point mutation leading to an exchange of a serine to tyrosine at codon 555. Because the position of stabilizing mutations would be more difficult to predict for the non-copy-up candidate 4, we sequenced the entire replicon. Two single-point mutations were found, one in the replication origin *oriII* (genomic position 213 according to NC_002506.1) and the other in the transcriptional terminator of the encoded *gfp* gene (Figure S3 in Supplementary Material). To derive the contribution of each of these mutations, we introduced them individually to an otherwise unchanged synVicII and measured respective replicon stabilities (Figure S4 in Supplementary Material). While the mutation at *gfp* slightly increased the stability compared to synVicII, the origin mutation actually decreased it. It appears that both mutations act synergistically to increase replicon stability as found in candidate 4. Certainly, further analyses are needed to understand the molecular basics of this finding.

### Probing the Origin Diversity of Secondary Chromosomes from *Vibrionaceae*

**Figure 3:**
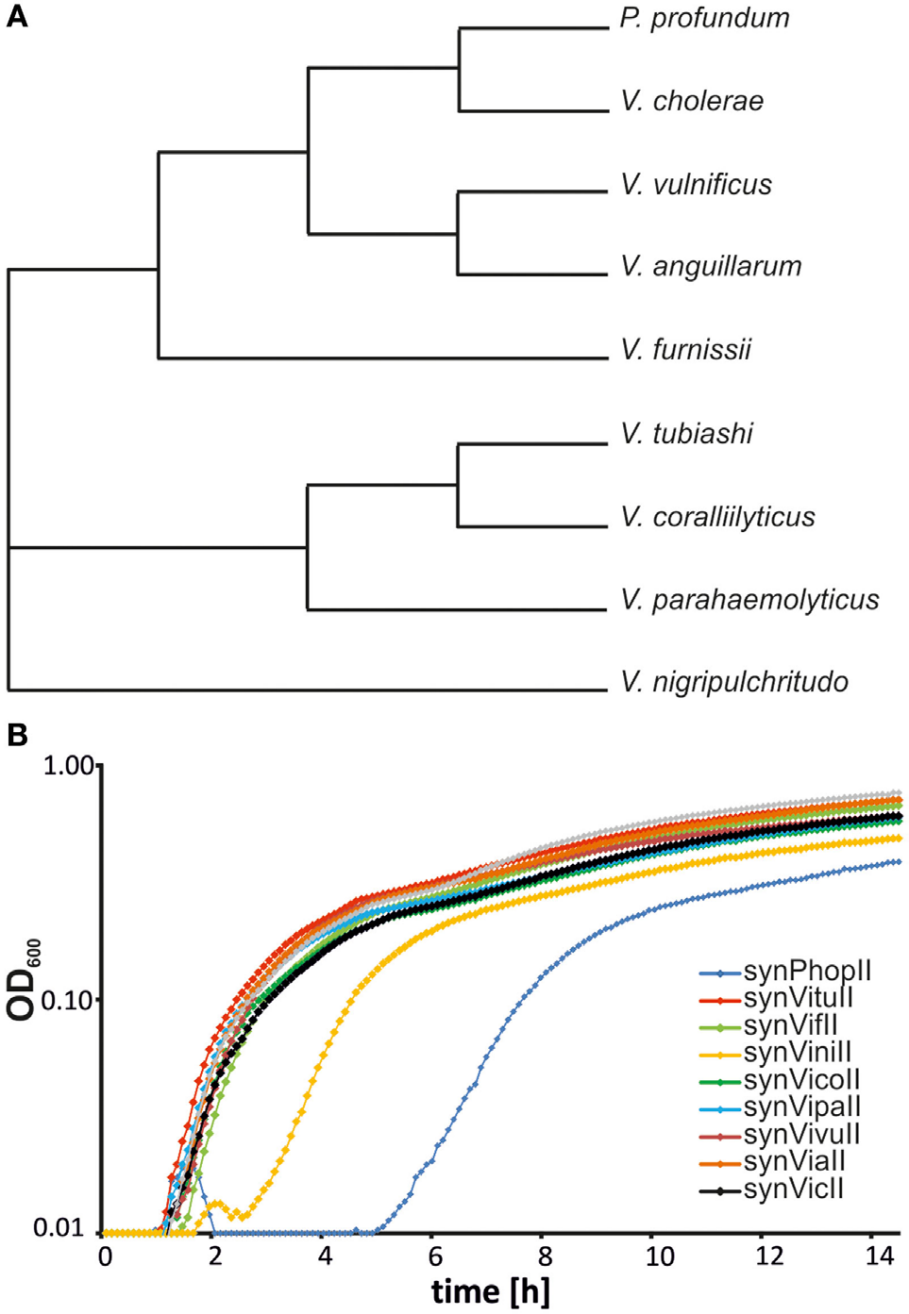
**Probing the origin diversity of secondary chromosomes from diverse *Vibrio***. **(A)** Phylogenetic tree of analyzed *Vibrionaceae* species based on 16S rRNA sequences. The respective alignment was calculated with EMBOS needle (Rice et al., 2000). 16S rRNA sequences (the one nearest the origin) were derived from the following genomes: NC_002505.1 for *Vibrio cholerae*, NC_006370.1 for *P. profundum*, NC_015633.1 for *V. anguillarum*, NC_016602.1 for *V. furnissii*, NC_004603.1 for *V. parahaemolyticus*, NC_005139.1 for *V. vulnificus*, NC_022528.1 for *V. nigripulchritudo*, NZ_CP009354.1 for *V. tubiashi*, and NZ_CP009264.1 for *V. coralliilyticus*. **(B)** Growth curves of *Escherichia coli* MG1655 strains carrying synthetic secondary chromosomes based on different *Vibrionaceae* replication origins. Strains used are SMS121 (synPhopII(AmpR)), SMS101 (synVituII(AmpR)), SMS102 (synVifII(AmpR)), SMS106 (synViniII(AmpR)), SMS107 (synVicoII(AmpR)), SMS108 (synVipaII(AmpR)), SMS110 (synVivuII(AmpR)), SMS134 (synViaII(AmpR)), and NZ72 (synVicII-1.352). Cells were grown in LB medium with ampicillin. OD_600_ was measured in 5-min intervals in a Victor X3 microplate reader.

If it is an attractive idea to have a synthetic secondary chromosome for biotechnology applications and basic research, the question occurs if it might also be interesting to have a third or fourth chromosome in addition. Spreading the genetic information to multiple replicons might actually have considerable benefits (Liang et al., 2013; Schindler and Waldminghaus, 2015; Milbredt et al., 2016). Since using the replication origin of the *V. cholerae* secondary chromosome as basis for a synthetic secondary chromosome in *E. coli* has proven a suitable approach, we set out to probe the origin diversity of the *Vibrio* genus for its potential as third chromosome. To this end, we constructed new replicons based on eight secondary replication origins derived from different *Vibrio* species and one *Photobacterium* (Figure 3A). The backbone was a newer version of synVicII including an origin of transfer (*oriT*) to allow conjugational replicon transfer (see following chapter and Materials and Methods). A prerequisite of having two replicons in a cell in addition to the primary chromosome is that they are not incompatible. Incompatibility is a long known phenomenon describing the observation that a plasmid is not kept in a cell that harbors a plasmid of the same ancestry (Scaife and Gross, 1962; Bouet et al., 2007). The molecular mechanisms underlying incompatibility can be different but are mostly related to replicon segregation and replication (Bouet et al., 2007). To measure the compatibility of replicons, we constructed additional versions with an alternative selection marker (kanamycin instead of ampicillin). All replicons were able to replicate in *E. coli*, and the growth of respective strains was relatively similar except variations of the lag-phase duration (Figure 3B). Longer lag-phases might indicate instability of the respective secondary chromosome. In an overnight culture the antibiotic will be inactive after some generations of growth, and the cells growing without selection pressure could lose an unstable replicon. Dilution of such a culture into fresh medium with antibiotic would then correspond to a lower number of resistant cells for cell growth as reflected by a long lag-phase. We performed crosswise conjugations in all possible combinations with replicons based on an F-plasmid origin as positive control. Except this control, none of the pairwise combination produced a significant amount of transconjugants (data not shown). We conclude that replicons based on replication origins from secondary chromosomes of the *Vibrio* genus and *Photobacterium* all belong to the same incompatibility group and are not suited for combination in one host cell. However, they all replicate within the heterologous host *E. coli* and could be used as alternative to synVicII in principle.

### New Version of synVicII

**Figure 4:**
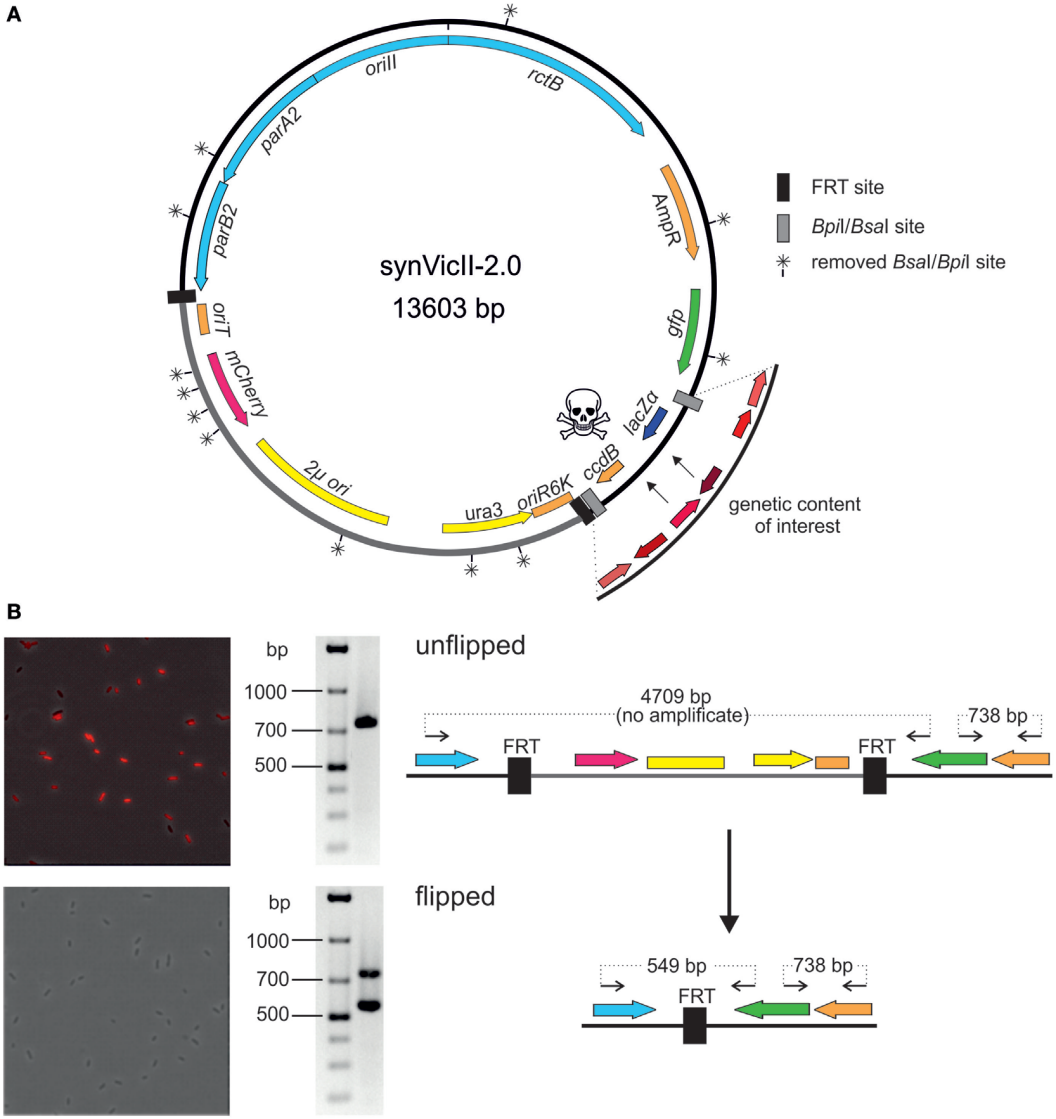
**New features of synthetic secondary chromosomes of type synVicII-2.0 (A) Scheme of synVicII-2.0** (for construction, see Materials and Methods). Genes are indicated by colored arrows and origins as blocks. *Bsa*I/*Bpi*I recognition sites were removed rendering synVicII suitable for modular cloning. Insertion of an origin of transfer allows conjugal transfer. For extension of the synVicII backbone, a *lacZ*α–*ccdB* cassette flanked by *Bsa*I or *Bpi*I recognition sites was inserted to use blue/white screening and *ccdB* toxicity in standard *Escherichia coli* strains for efficient detection of recombinant DNA (Bernard and Couturier, 1992). Some parts of the replicon are only needed for efficient assembly and transfer as, for example, the yeast replication origin and selection marker. Flanking the respective region by FRT sites allows removal after successful assembly by flippase-based site-specific recombination (Cherepanov and Wackernagel, 1995). **(B)** The red fluorescence reporter under the control of the P*_lac_* promoter allows easy readout of successful recombinations as shown by fluorescence microscopy and PCR analysis of unflipped (top panel) and flipped (bottom panel) as illustrated in the right panel. Data shown are for strain NZ67 carrying synVicII-1.34 transformed with pCP20 at 30°C to activate FRT recombination. Upon heat induction, the heat sensitive replicon pCP20 got lost. Colony PCR with primers (back arrows in right panel) 716/24 and 858/25 also confirmed successful flipping. Elongation time was short enough to allow amplification of maximum 800 bp, and expected fragment sizes are indicated in the right panel.

Well-characterized replicons are a prerequisite for solid genetic work in basic research and biotechnology. We had previously developed a prototype of the synthetic secondary chromosome synVicII (Messerschmidt et al., 2015). Meanwhile, we have introduced several innovations as summarized in Figure 4A into a new version synVicII-2.0. A first change to the previous synVicII is the introduction of an *oriT* to allow transfer *via* conjugation. This feature is especially important for larger replicons because efficiencies of isolation and transformation drop with replicon size (Gowland and Hardman, 1986; Sheng et al., 1995). We have successfully tested the transfer of synVicII-2.0 versions with different inserts from a donor strain carrying the conjugation machinery to wild-type *E. coli* cells (data not shown). A second new feature of synVicII-2.0 is the possibility to excise a region of the replicon that is needed only for the construction process. This region includes the conditional replication origin *ori*R6K, the yeast marker and replication origin, and *oriT* (Figure 4A). The excision is mediated by two flanking FRT recombination sites, and a simple readout of successful loss of this region is possible through an inserted *mCherry* reporter gene (Figure 4B). Removing this construction region is likely to limit interference with the genetic content of interest. The third change to synVicII was its conversion into a modular cloning (MoClo)-compatible replicon. MoClo is an assembly framework based on type IIs restriction enzymes (Weber et al., 2011; Werner et al., 2012). The MoClo system is now widely used with still increasing popularity (Engler et al., 2014; Kakui et al., 2015; Schindler et al., 2016). It consists of vector sets (level 0, level 1, level M, level P) with the 4-bp overhang of each vector matching the overhangs of the preceding and following vector, respectively. Assembling multiple fragments into an acceptor vector is possible because the resistance markers as well as the type IIs restriction sites are alternating. Assemblies of different numbers of fragments are facilitated by a set of specific endlinkers. To make the benefits of the MoClo system accessible for synVicII engineering, we removed all 12 *Bpi*I and *Bsa*I restriction sites by a two-step multi-fragment assembly in yeast (Figure 4A; see Materials and Methods for details). In addition, we introduced level M or level P MoClo cassettes consisting of the suicide gene *ccdB* and the reporter *lacZ* flanked by either *Bpi*I or *Bsa*I sites [Figure 4A; Schindler et al. (2016)]. This resulted in 14 synVicII backbones with full compatibility to the MoClo system (Weber et al., 2011; Werner et al., 2012). Insertion of genetic content of interest will remove the *ccdB–lacZ* cassette generating viable white colonies. Because the synVicII backbone as well as the endlinker plasmids possess an ampicillin resistance marker, the marker of all 14 level M and P endlinker plasmids was changed to chloramphenicol (Table S2 in Supplementary Material).

**Figure 5:**
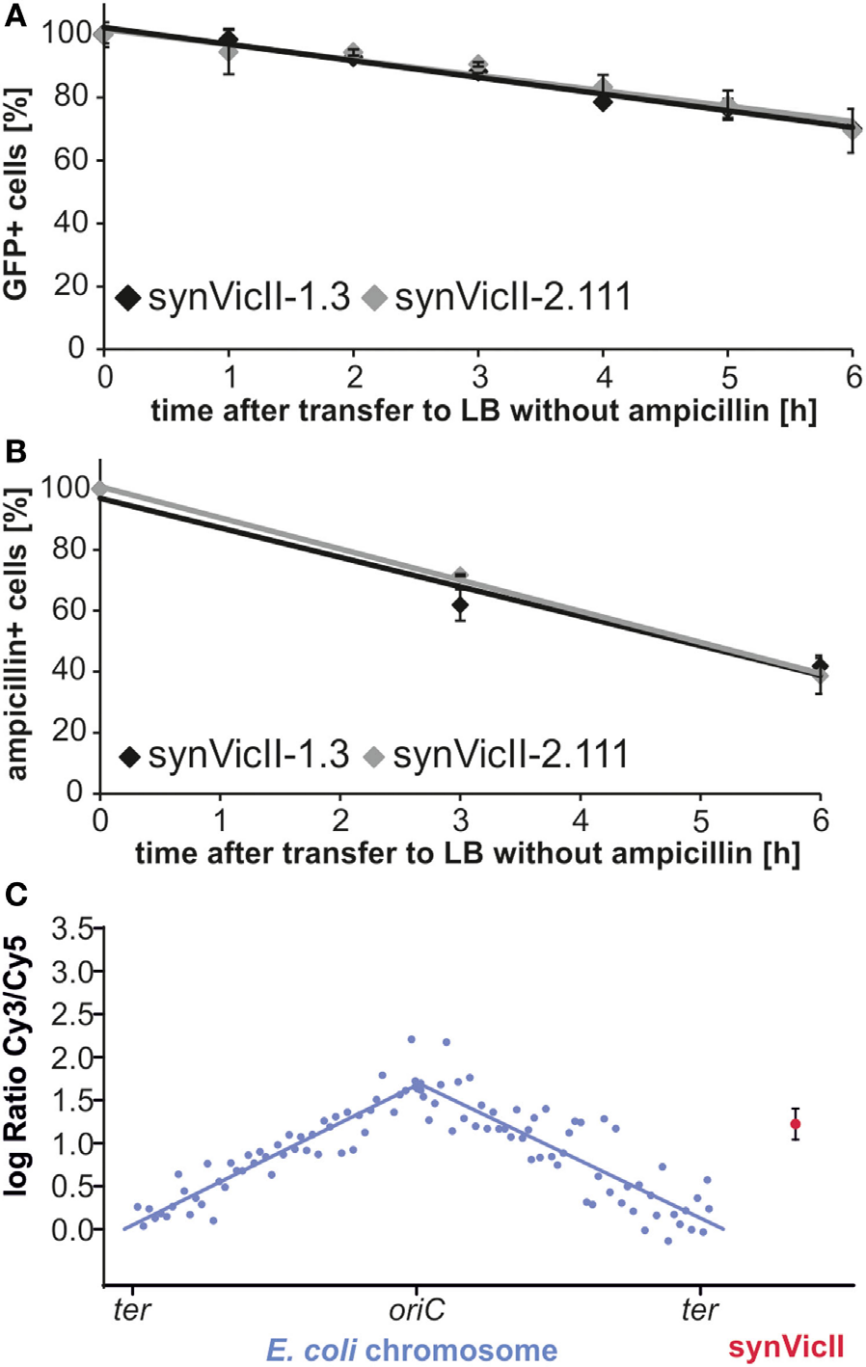
**Conserved genetic characteristics of new synVicII**. **(A)** Stability of synVicII-1.3 (black) and synVicII-2.111 (gray) in *Escherichia coli* MG1655 determined by flow cytometry measured as described (Messerschmidt et al., 2015). Strains carrying synVicII-1.3 (SMS18) or synVicII-2.111 (DS292) were grown in LB supplemented with ampicillin and 0.2 mM IPTG at 37°C to an OD_600_ of about 0.15, transferred to ampicillin-free medium and cultured for 6 h. Cultures were kept in exponential phase by diluting into fresh pre-warmed medium at an OD_600_ higher than 1.2. Samples were taken every 1 h and *gfp* fluorescence as proxy for replicon presence measured by flow cytometry. **(B)** Stability of synVicII-1.3 (black) compared to synVicII-2.111 (gray) in *E. coli* MG1655 measured by counting ampicillin-resistant colonies after transfer to medium without antibiotic selection at indicated time points (Messerschmidt et al., 2015). Results are from 3 biological replicates with a total of 300 colonies per time point and strain. **(C)** Comparative genomic hybridization of *E. coli* strain DS292 harboring synVicII-2.111. DNA of exponentially grown DS292 was hybridized against a control with fully replicated chromosomes (see Materials and Methods for details). Respective logarithmic values of ratios are plotted against their genomic position. Blue dots represent the 104 probes for the *E. coli* chromosome, blue lines the linear curve fitted to chromosome arms. The red dot represents the mean of the three probes of synVicII with the corresponding standard deviation.

To test if the new version of synVicII retains its previous characteristics, we constructed an “empty” replicon by performing a MoClo reaction with synVicII-2.11 and the respective endlinker pMA678 only, since the cloning cassette would kill wild-type cells (=synVicII-2.111, Table S2 in Supplementary Material). This replicon showed a very similar stability within *E. coli* cells compared to the original synVicII-1.3 as measured by flow cytometry and a colony counting approach as previously described [Figures 5A,B; Messerschmidt et al. (2015)]. The observed instability might be explained by recent studies showing that *oriII* activation is triggered by replication of a short site (*crtS*) on chromosome I in *V. cholerae* (Baek and Chattoraj, 2014; Val et al., 2016). Trans activation of *oriII* firing by *crtS* is obviously not essential in the heterologous host *E. coli* because synVicII replicates without a *crtS* site, which is absent from the primary *E. coli* chromosome. However, it is reasonable to assume that insertion of a *crtS* site into *E. coli* could stabilize synVicII within the *E. coli* cells. Respective studies could in turn help to understand the underlying mode of action for the *crtS–oriII* system.

Comparative genomic hybridization analysis showed that the synVicII-2.111 copy number lies between the copy number of the replication origin and terminus of the primary chromosome as shown for the original synVicII-1.3 (Figure 5C) (Messerschmidt et al., 2015). We conclude that the new version of synVicII remains the previously established genetic characteristics despite the introduced changes.

## Materials and Methods

### Bacterial Strains, Plasmids, Oligonucleotides, and Culture Conditions

Strains, plasmids, and oligonucleotides are listed in Tables S1–S3 in Supplementary Material. Pre-cultures of *E. coli* were grown in 3 ml LB medium. Antibiotics and inductors were used with the following concentrations if not indicated otherwise: ampicillin (100 µg/ml), kanamycin (100 µg/ml), chloramphenicol (35 µg/ml), and IPTG (100 µg/ml). Cultures of *S. cerevisiae* were as described previously (Messerschmidt et al., 2015).

### Construction of Secondary Chromosomes and Plasmids

All replicons were constructed by Gibson assembly and reactions transformed into *E. coli* XL-1 Blue or *E. coli* DH5α λ *pir* if not indicated otherwise (Gibson et al., 2009). The previously published synVicII-1.3 was changed stepwise toward synVicII-2.0 as follows. An *Xho*I recognition site was inserted by Gibson assembly of a PCR product with primers 327/328 from synVicII-1.5 (Messerschmidt et al., 2015) and *Nru*I-digested synVicII-1.3, resulting in synVicII-1.31. For the construction of synVicII-1.32, the *I-Sce*I site in synVicII-1.31 was replaced with a *Pvu*II site. For that purpose *I-Sce*I-digested synVicII-1.31 and oligonucleotide 814 were assembled by homologous recombination in *S. cerevisiae* strain pJ69-4a as described (Colot et al., 2006; Gietz and Schiestl, 2007). For the construction of synVicII-1.311, the RhaT promotor was amplified with primers 329/330 from pWBT5 (Schlüter et al., 2015). The forward primer has an overhang adding one FRT site. The fragment was integrated into *Xho*I-cut synVicII-1.31. To generate synVicII-1.312, *rfp* was amplified with primers 331 and 597 from pSB1C3 J04450 (iGEM). The reverse primer has an overhang adding one FRT site. The PCR product was then assembled with *Nhe*I-digested synVicII-1.311.

To generate synVicII-1.33, the FRT site with *Sma*I and the FRT site with *I-Sce*I recognition site were amplified with primers 817/818 and 815/816 from synVicII-1.312 and integrated into *I-Sce*I-cut synVicII-1.32. synVicII-1.34 was made by amplification of *rfp* with primers 819/820 from pSB1C3J04450 (iGEM) and integration into *Sma*I-digested synVicII-1.33. synVicII-1.35 was constructed by combining PCR-amplified *oriT* (primers 874 and 875 from pUC18-R6KT-egfp) and *Sma*I-digested synVicII-1.34. To construct synVicII-1.36, *lacZ* and *ccdB* were amplified with oligonucleotides 1002 and 1005 from pMA58 (Schindler et al., 2016). Genes were integrated into *I-Sce*I-digested synVicII-1.35.

The mutation of *Bpi*I and *Bsa*I recognition sites within synVicII-1.36 was made by cutting the replicon with one of the enzymes and transforming the fragments into yeast together with bridging DNA fragments changing the respective sites. Bridging DNA for the mutation of four *Bpi*I and two *Bsa*I recognition sites was generated by designing pairs of 60 bp oligonucleotides with 20 bp annealing region (primer pairs: 1628–1635 and 1638–1641). The resulting 100 bp DNA fragments were generated by a 3-cycle PCR with respective primer pairs. Additional four *Bpi*I sites were deleted by replacing *rfp* with an optimized *mCherry* amplified with primer pair 1636 and 1637 from pMA17. pMA17 was generated by a MoClo reaction into pICH41276 using two PCR products to remove a recognition site with primer pairs 69 and 70 and 71 and 72 from template pWBT5*^mCherry^*. One *Bsa*I site was mutated by amplifying *bla* from pMA53 with primer 214 and 215. synVicII-1.36 was cut with *Bpi*I or *Bsa*I and transformed together with the corresponding DNA parts into *S. cerevisiae* VL6-48N to produce either *Bpi*I recognition site-free synVicII-1.361 or *Bsa*I recognition site-free synVicII-1.362 by *in vivo* homologous recombination. For each construct, *S. cerevisiae* colonies were pooled, cultivated in 50 ml SD-ura, and plasmid DNA extracted. The plasmid DNA was digested with *Bpi*I or *Bsa*I to remove false positives and subsequently transformed into *E. coli* DB3.1 λ pir. Positive clones were verified by restriction analysis. DNA of synVicII-1.361 and synVicII-1.362 was pooled in equimolar concentration, digested with *Bpi*I and *Bsa*I, and transformed into *S. cerevisiae* to generate *Bpi*I and *Bsa*I recognition site-free synVicII-1.37. In order to generate MoClo-compatible level M and level P backbones, synVicII-1.37 was cut with *Not*I and transformed in 14 reactions with the respective 7 level M and 7 level P MoClo cassettes to generate synVicII-2.01 to synVicII-2.07 and synVicII-2.11 to synVicII-2.17, respectively. Corresponding level M and P MoClo cassettes were amplified using primer 1029 and 1030 and templates pMA60–pMA66, respectively, primer 1031 and 1032 and templates pMA67–pMA73 (Schindler et al., 2016).

The existing MoClo endlinker of the Marillonnet group possess *bla* and interfere with the MoClo synVicII-2.0 backbones. Therefore, the *bla* gene was exchanged with *cat*. To this end, level M and P endlinker plasmids were amplified with primer pair 582 and 1099 and the *cat* gene with primer pair 581 and 1100 from pMA44 (Daniel Schindler, unpublished) resulting in plasmids pMA667–680.

synVicII-1.301 was constructed by inserting PCR-amplified *oriC* without one of the 13mers from gDNA of strain SMS18 into *I-Sce*I-digested synVicII-1.3. To generate synVicII-1.302, part of *lacZ* was amplified with primers 876 and 877 from gDNA of strain SMS18 and integrated into *I-Sce*I-digested synVicII-1.3.

synVicII-0.11 was constructed by assembling *gfp*-AAV amplified with primers 28/29 from synVicII-1.3 with *I-Sce*I-digested synVic-0.1 by homologous recombination in yeast. For construction of synVicII-1.313, the *oriII* of the selection candidate 4 (synVicII-1.8) was amplified with primers 14/16 and assembled with *Not*I-digested synVicII-0.11 by homologous recombination in yeast. The *gfp* from synVicII-1.8 was amplified with primers 26/27 and integrated into *I-Sce*I-digested synVicII-1.0 by Gibson assembly resulting in synVicII-1.314.

For construction of synthetic secondary chromosomes based on different *Vibrio* genomes, *oriII*s with *parAB* and *rctB* were amplified from gDNA of the respective strain. gDNA was isolated with the phenol–chloroform method as described in Schindler et al. (2016). To facilitate origin cloning, the *oriII* in synVicII-1.35 was replaced with *lacZ*α. For this construction, *lacZ*α was amplified with primers 1132/1133 and assembled with *Eco*RI-*Sal*I-digested synVicII-1.35 by yeast homologous recombination. All oligonucleotides for *oriII* cloning have fitting overhangs to the neighboring fragment in the backbone synVicII-1.351 (at least 25 bp) allowing the construction with Gibson assembly and add an *Asc*I site to allow *oriII* release.

To construct synPhopII(AmpR), the *oriII* region was amplified with primers 1148/1149 from gDNA of *P. profundum* and assembled with *Asc*I-digested synVicII-1.351. synVituII(AmpR), synVifII(AmpR), synViniII(AmpR), synVicoII(AmpR), synVipaII(AmpR), synVivuII(AmpR), and synViaII(AmpR) were constructed accordingly with respective primers and templates listed in Table S4 in Supplementary Material.

To exchange the ampicillin resistance marker in synVicII-1.3 with a kanamycin resistance marker, *kan* was amplified with primer 30/31 from pUC57 and assembled with *Bgl*I-digested synVicII-1.3 by homologous recombination in yeast to generate synVicII-1.7.

For the construction of other kanamycin-resistant replicons, the backbone synVicII-0.3 with *kan* was generated by religation of *Asc*I-digested synVicII-1.351 (= synVicII-1.3511) followed by cutting with *Bgl*I. This linearized fragment was assembled with the kanamycin cassette amplified with primers 1435/31 from synVicII-1.7 by homologous recombination in yeast as described above. For synPhopII(*kan*), *Asc*I-digested synVicII-0.3 was ligated with the *Asc*I-digested *oriII* part of synPhopII(AmpR), the corresponding ampicillin-resistant replicon. synVituII(*kan*), synVifII(*kan*), synViniII(*kan*), synVicoII(*kan*), synVipaII(*kan*), synVivuII(*kan*), and synViaII(*kan*) were constructed accordingly. synF-2.0 was constructed the same way with F *ori* amplification from synF-plasmid with primers 1487/1488.

### Stable synVicII Selection Experiments

*Escherichia coli* strain SMS18 carrying synVicII-1.3 was grown overnight in LB medium with ampicillin and was then 1:1,000 diluted in LB medium without antibiotics. After 8 h of growth, cells were transferred 1:10,000 into LB with ampicillin and grown overnight. The procedure was repeated for 3 days and finally 100 µl of culture plated on selective plates. Replicons of individual clones were isolated and retransformed into *E. coli* MG1655. Replicon stability was measured as before (Messerschmidt et al., 2015). To select for non-copy-up and copy-up mutants, candidates were grown in LB medium with either 100 or 1,500 µg/ml ampicillin in a 96-well plate in a microplate reader (Victor X3 Multilabel Plate Reader, PerkinElmer) at 37°C. The 150 µl of main culture was inoculated 1:1,000, covered with 70 µl of mineral oil, and growth curves recorded for 14.5 h.

### Comparative Genomic Hybridization

Microarray construction, sample preparation, hybridization, and data processing were essentially performed as described (Messerschmidt et al., 2015). Instead of harvesting cells in stationary phase for a hybridization control, exponentially growing cells of strain FSK18 were treated with 150 µg/ml rifampicin for 2 h. Lysed cells were treated with 60 µg/ml RNase A for 1 h at 65°C before DNA isolation with phenol–chloroform and ethanol precipitation.

### Southern Blot Experiments

For Southern hybridization, genomic DNA was extracted from 1.5 ml culture at an OD_600_ of 0.3 as described with the following minor changes (Skarstad and Lobner-Olesen, 2003). Treatment in the DNA isolation buffer was performed at 4°C, and the RNase A incubation was for 1 h. After phenol–chloroform extraction, DNA was precipitated with ethanol and Na-acetate. For blotting, usually 1 µg of *Nco*I-digested chromosomal DNA was separated on 1% agarose gels and transferred by vacuum blotting to an Amersham Hybond-N membrane (GE Healthcare, Chalfont St Giles). Exceptions with other amount of DNA are mentioned in the figure legends (SMS67 and the replicon controls synVicII-1.3/synEsc-1.3). DNA was detected with a DIG labeled AmpR probe (PCR DIG Probe Synthesis Kit, Roche, Penzberg) as PCR product from primers 793/794 with synVicII-1.3 as a template.

## Author Contributions

SM, DS, CZ, FK, and NS performed the experiments. SM, DS, CZ, FK, NS, and TW analyzed the data. SM, DS, FK, and TW made the figures and wrote the manuscript.

## Conflict of Interest Statement

The authors declare that the research was conducted in the absence of any commercial or financial relationships that could be construed as a potential conflict of interest.
