# Supplementary material for: Optimization and Characterization of the Synthetic Secondary Chromosome synVicII in Escherichia coli
Source: Front Bioeng Biotechnol. 2016 Dec 23;4:96. doi: 10.3389/fbioe.2016.00096 (PMC5179572; doi:10.3389/fbioe.2016.00096)
Supplement: Supplementary file 1 [file Presentation_1.PDF]

Supplementary Material

Supplementary tables S1-S5

Table S1: Strains used in this work

Strain	Relevant genotype	Reference
E. coli DH5*α λ pir*	*supE*44, Δ*lacU*169 (Φ*lacZ*ΔM15), *recA*1, *endA*1, *hsdR*17, *thi*-1, *gyrA*96, *relA*1, λ *pir* phage lysogen	(Miller and Mekalanos, 1988)
E. coli DB3.1 λpir	F- *gyrA*462 *endA*1 *glnV*44 Δ(sr1-*recA*) *mcrB mrr hsdS*20(rB-, mB-) *ara*14 *galK*2 *lacY*1 *proA*2 *rpsL*20 *xyl*5 Δ*leu mtl*1, λ *pir* phage lysogen	(House et al., 2004)
E. coli XL1 Blue	*supE*44, *hsdR*17, *recA*1, e*ndA*1, *gyrA*96, *thi1, relA*1, *lac*‾ [F' *proAB, lac*^{q}*Z*M15, Tn10(tet^{r})]	Stratagene, Agilent Technologies, Germany
E. coli MG1655	wild type	(Blattner et al., 1997)
E. coli WM3064	Donor strain for conjugation: *thrB1004 pro thi rpsL hsdS lacZ*ΔM15 RP4-1360 Δ(*araBAD*)*567* Δ*dapA1341*::[*erm pir*(wt)]	William Metcalf
V. cholerae El Tor N16961	StrR	(Heidelberg et al., 2000)
Vibrio tubiashii		DSMZ Braunschweig No. 19142
Vibrio nigripulchritudo		DSMZ No. Braunschweig 21607
Vibrio corallilyticus		DSMZ No. Braunschweig 19607
Vibrio anguillarum		DSMZ Braunschweig No. 21597
Vibrio parahaemolyticus		DSMZ Braunschweig No. 10027
Vibrio furnissii		DSMZ Braunschweig No. 14383
Vibrio vulnificus		DSMZ Braunschweig No. 10143
Photobacterium profundum		DSMZ Braunschweig No. 21095
S. cerevisiae PJ69-4a	MATa, *trp*1-901, *leu*2-3, *leu*2-112, *ura*-3-52, *his*3-200, *gal4*Δ, *gal80*Δ, *gal*2-*ade*2, *lys*2::*gal*1*his3*, *met*2::*gal*7-	(James et al., 1996)
S. cerevisiae VL6-48N	MATα *trp*1-Δ1 *ura*3-Δ1 *ade*2-101 *his*3-Δ200 l*ys*2 *met*14 cir°	(Kouprina et al., 1998)
DS292	*E. coli* MG1655 synVicII-2.111	This work
SMS18	*E. coli* MG1655 synVicII-1.3	(Messerschmidt et al., 2015)
SMS65	*E. coli* MG1655 synEsc-1.31	This work
SMS66	*E. coli* MG1655 Δ*seqA* synVicII-1.3	This work
SMS67	*E. coli* MG1655 Δ*seqA* synEsc-1.31	This work

Strain	Relevant genotype	Reference
SMS72	*E. coli* MG1655 synVicII-1.301	This work
SMS74	*E. coli* MG1655 synVicII-1.302	This work
SMS121	*E. coli* MG1655 synPhopII(amp)	This work
SMS101	*E. coli* MG1655 synVituII(amp)	This work
SMS102	*E. coli* MG1655 synVifII(amp)	This work
SMS106	*E. coli* MG1655 synViniII(amp)	This work
SMS107	*E. coli* MG1655 synVicoII(amp)	This work
SMS108	*E. coli* MG1655 synVipaII(amp)	This work
SMS110	*E. coli* MG1655 synVivuII(amp)	This work
SMS134	*E. coli* MG1655 synViaII(amp)	This work
NZ72	*E. coli* MG1655 synVicII-1.352	This work

Table S2: Plasmids used in this work

Plasmid	Relevant characteristics[a)]	Reference
pICH41276	MoClo Level 0 Plasmid	(Weber et al., 2011)
pICH50872	MoClo Level M endlinker	(Weber et al., 2011)
pICH50881	MoClo Level M endlinker	(Weber et al., 2011)
pICH50892	MoClo Level M endlinker	(Weber et al., 2011)
pICH50900	MoClo Level M endlinker	(Weber et al., 2011)
pICH50914	MoClo Level M endlinker	(Weber et al., 2011)
pICH50927	MoClo Level M endlinker	(Weber et al., 2011)
pICH50932	MoClo Level M endlinker	(Weber et al., 2011)
pICH79255	MoClo Level P endlinker	(Weber et al., 2011)
pICH79264	MoClo Level P endlinker	(Weber et al., 2011)
pICH79277	MoClo Level P endlinker	(Weber et al., 2011)
pICH79289	MoClo Level P endlinker	(Weber et al., 2011)
pICH79290	MoClo Level P endlinker	(Weber et al., 2011)
pICH79300	MoClo Level P endlinker	(Weber et al., 2011)
pICH79311	MoClo Level P endlinker	(Weber et al., 2011)
pMA17	pICH41276 *mCherry*	This work
pMA53	Level 1 MoClo plasmid	(Schindler et al., 2016)
pMA58	Level 1 MoClo Plasmid	(Schindler et al., 2016)
pMA60	MoClo Level M 1	(Schindler et al., 2016)
pMA61	MoClo Level M 2	(Schindler et al., 2016)

Plasmid	Relevant characteristics[a)]	Reference
pMA62	MoClo Level M 3	(Schindler et al., 2016)
pMA63	MoClo Level M 4	(Schindler et al., 2016)
pMA64	MoClo Level M 5	(Schindler et al., 2016)
pMA65	MoClo Level M 6	(Schindler et al., 2016)
pMA66	MoClo Level M 7	(Schindler et al., 2016)
pMA67	MoClo Level P 1	(Schindler et al., 2016)
pMA68	MoClo Level P 2	(Schindler et al., 2016)
pMA69	MoClo Level P 3	(Schindler et al., 2016)
pMA70	MoClo Level P 4	(Schindler et al., 2016)
pMA71	MoClo Level P 5	(Schindler et al., 2016)
pMA72	MoClo Level P 6	(Schindler et al., 2016)
pMA73	MoClo Level P 7	(Schindler et al., 2016)
pMA667	MoClo Level M endlinker	This work
pMA668	MoClo Level M endlinker	This work
pMA669	MoClo Level M endlinker	This work
pMA670	MoClo Level M endlinker	This work
pMA671	MoClo Level M endlinker	This work
pMA672	MoClo Level M endlinker	This work
pMA673	MoClo Level M endlinker	This work
pMA674	MoClo Level P endlinker	This work
pMA675	MoClo Level P endlinker	This work
pMA676	MoClo Level P endlinker	This work
pMA677	MoClo Level P endlinker	This work
pMA678	MoClo Level P endlinker	This work
pMA679	MoClo Level P endlinker	This work
pMA680	MoClo Level P endlinker	This work
pUC57kan	Cloning vector, *kan*	GenScript, Piscataway Township, NJ, USA
pWBT5^{mCherry}	P_{rhaT}	(Schlüter et al., 2015)
synEsc-1.3	synX-0.1 + *oriC* (coord. 3923616-3924064)	(Messerschmidt et al., 2015)
synEsc-1.31	synEsc-1.3 with *mioC*	This work
synF-2.0	synVicII-0.3+ F $origin_{synF\text{-}plasmid}$	This work
synF-plasmid	synX-0.3 with F *origin*, *sopABC*	(Messerschmidt et al., 2015)
synPhopII(AmpR)	synVicII-1.351 + $oriII_{P.\ profundum}$ (coord. 2234799-2736)	This work
synPhopII(*kan*)	synVicII-0.3+ $oriII_{P.\ profundum}$	This work

Plasmid	Relevant characteristics[a)]	Reference
synViaII(AmpR)	synVicII-1.351 + *oriII*$_{V.\ anguillarum}$ (coord.	This work
synViaII(*kan*)	synVicII-1.351 + *oriII*$_{V.\ anguillarum}$	This work
synVicII-0.1	*rctB* (coord. 1134–3110), *parAB* (coord. 1070018-1072220), *ori*R6K; *2µ ori*, *ura3*, AmpR	(Messerschmidt et al., 2015)
synVicII-0.11	synVicII-0.1 + *gfp*(AAV)	This work
synVicII-0.3	synVicII-1.351 AmpR::*kan*	This work
synVicII-1.0	synVicII 0.1 + *oriII* (coord. 1072221-1133)	(Messerschmidt et al., 2015)
synVicII-1.3	synVicII-1.0 + $P_{A1/04/03}$-RBSII-*gfp*(AAV)	(Messerschmidt et al., 2015)
synVicII-1.301	synVicII-1.3 + *oriC* w/o 13mer (coord. 3923811-3924568)	This work
synVicII-1.302	synVicII-1.3 + *lacZ* (coord. 363541-364605)	This work
synVicII-1.31	synVicII-1.3 + *Xho*I between *2µ ori* and *parB*	This work
synVicII-1.311	synVicII-1.31 + P_{rhaT} in *Xho*I	This work
synVicII-1.312	synVicII-1.311 + *rfp* in *Nhe*I	This work
synVicII-1.313	synVicII-0.11 + *oriII*$_{\text{candidate 4 evolution}}$	This work
synVicII-1.314	synVicII-1.0 + *gfp*$_{\text{candidate 4 evolution}}$	This work
synVicII-1.32	synVicII-1.31 Δ*I-Sce*I, +*Pvu*II	This work
synVicII-1.33	synVicII-1.32 + FRT site *Sma*I + FRT site *I-Sce*I	This work
synVicII-1.34	synVicII-1.33 + *rfp*	This work
synVicII-1.35	synVicII-1.34 + *oriT*	This work
synVicII-1.351	synVicII-1.35, *oriII*$_{V.cholerae}$::*lacZα*	This work
synVicII-1.3511	synVicII-1.351 Δ*lacZα*	This work
synVicII-1.36	synVicII-1.35 + *lacZα* + *ccdB*	This work
synVicII-1.361	synVicII-1.36 w/o *Bpi*I recognition sites	This work
synVicII-1.362	synVicII-1.36 w/o *Bsa*I recognition sites	This work
synVicII-1.37	synVicII-1.36 w/o *Bpi*I and *Bsa*I recognition sites	This work
synVicII-1.7	synVicII-1.3 AmpR::*kan*	
synVicII-1.8	synVicII-1.3 selection candidate 4	This work
synVicII-1.9	synVicII-1.3 selection candidate 5	This work
synVicII-1.10	synVicII-1.3 selection candidate 3	This work
synVicII-2.01	synVicII-1.37 MoClo Level M 1 (pMA60)	This work
synVicII-2.02	synVicII-1.37 MoClo Level M 2 (pMA61)	This work
synVicII-2.03	synVicII-1.37 MoClo Level M 3 (pMA62)	This work
synVicII-2.04	synVicII-1.37 MoClo Level M 4 (pMA63)	This work
synVicII-2.05	synVicII-1.37 MoClo Level M 5 (pMA64)	This work
synVicII-2.06	synVicII-1.37 MoClo Level M 6 (pMA65)	This work
synVicII-2.07	synVicII-1.37 MoClo Level M 7 (pMA66)	This work
synVicII-2.11	synVicII-1.37 MoClo Level P 1 (pMA67)	This work
synVicII-2.111	synVicII-2.11 and pMA678 endlinker	This work
synVicII-2.12	synVicII-1.37 MoClo Level P 2 (pMA68)	This work
synVicII-2.13	synVicII-1.37 MoClo Level P 3 (pMA69)	This work
synVicII-2.14	synVicII-1.37 MoClo Level P 4 (pMA70)	This work
synVicII-2.15	synVicII-1.37 MoClo Level P 5 (pMA71)	This work
synVicII-2.16	synVicII-1.37 MoClo Level P 6 (pMA72)	This work
synVicII-2.17	synVicII-1.37 MoClo Level P 7 (pMA73)	This work
synVicoII(AmpR)	synVicII-1.351 + *oriII*$_{V.\ coralyticus}$ (coord. 1636573-	This work

Plasmid	Relevant characteristics[a)]	Reference
	1642380)	
synVicoII(*kan*)pMA893	synVicII-0.3+ *oriII*$_{V.\ coralyticus}$	This work
synVifII(AmpR)	synVicII-1.351 + *oriII*$_{V.\ furnissii}$ (coord. 1027513-1033467)	This work
synVifII(*kan*)	synVicII-0.3+ *oriII*$_{V.\ furnissii}$	This work
synViniII(AmpR)	synVicII-1.351 + *oriII*$_{V.\ nigripulchritudo}$ (coord.2212141-5725)	This work
synViniII(*kan*)	synVicII-0.3+ *oriII*$_{V.\ nigripulchritudo}$	This work
synVipaII(AmpR)	synVicII-1.351 + *oriII*$_{V.\ parahaemolyticus}$ (coord. 1874837-3408)	This work
synVipaII(*kan*)	synVicII-0.3+ *oriII*$_{V.\ parahaemolyticus}$	This work
synVituII(AmpR)	synVicII-1.351 + *oriII*$_{V.tubiashi}$ (coord. 1764492-3576)	This work
synVituII(*kan*)	synVicII-0.3+ *oriII*$_{V.tubiashi}$	This work
synVivuII(AmpR)	synVicII-1.351 + *oriII*$_{V.\ vulnificus}$ (coord.1853576-2515)	This work
synVivuII(*kan*)	synVicII-0.3+ *oriII*$_{V.\ vulnificus}$	This work

[a)] Genomic positions are indicated according to the following genome annotations: NC_002506.1 for ChrII of *V. cholerae,* NC_ 000913.2 for *E. coli* MG1655, for ChrII of *P. profundum* NC_00637.1, *V. anguillarum* NC_015637.1, *V. furnissii* NC_016628.1, *V. parahaemolyticus* NC_004605.1; *V. vulnificus* NC_005140.1, *V. nigripulchritudo* NC_022543.1, *V. tubiashi* NZ_CP009355.1 and *V. coralliilyticus* NZ_CP009265.1

Table S3: Oligonucleotides used in this work

Name	Sequence from 5' to 3'
14	CAATCTCAATTCGATCGGCCTGCACT
16	GGTTAGATCCGTATCACACTTACCGT
26	CTTAACGTGAGTTTTCGTTCCACTAGGGATAACAGGGTAATTTTATCAAAAAGAGTGTTGACTTGTGAG
27	GTACGTGAAACATGAGAGCTTAGTACGTACTATCAACAGGTTCCAAGCTAGCTTGGATTCTCAC
28	GATAGGTGCCTCACTGATTAAG
29	GGCTCTAAGGGCTTCTCAGT
30	GATTTGGCAAAATCCTGACTCAGTGCTTTCTATCCCGTGCAGCTCTGGCCCGTGTCT
31	CAATCTAAAGTATATATGAGTAAACTTGGTCTGACAGTTAGAAAAACTCATCGAGCATCAAATGAAAC
69	TGAAGACATGCTTGATGAGGATCTGCAGAGGAG
70	TGAAGACTAGGTTTTCTTCTGCATTACGGGGC
71	TGAAGACATAACCATGGGCTGGGAGGCCT
72	TGAAGACATAGCGACGGCCAGTGCCAAGCT
214	CGCTCATGAG ACAATAACCC
215	GCCTTCTTGA CGAGTTCTTC TGACTGTCAG ACCAAGTTTA CTCATATA
327	CGTTGGCAGCGCCTAAGAAACCAATAAGGCTAAGCCCTCGAGCCCTAAAACGCACAAAGCCC
328	TCAATGCTGAGCACGCTAAGTTT
329	GATGCGGGCTTTGTGCGTTTTAGGGCTCGAGGTACTTTTCGTAAGGGTATGG
330	CTAAGAAACCAATAAGGCTAAGCCCGAAGTTCCTATACTTTCTAGAGAATAGGAACTTCCTCGAGCATCTCCGACGAGATGAGT
331	GTACGTACTATCAACAGGTTCCAAGGAAGTTCCTATTCTCTAGAAAGTATAGGAACTTCGCTAGCAGAGGAGAAATTAAGCATGGCT TCCTCCGAAGACGT
581	GAGTAAACTTGGTCTGACAGTCATCGCAGTACTGTTGTATTCATTAAGC
582	CTGTCAGACCAAGTTTACTCATATATACTTTAGATTG
597	CGTTTTTTATTGGTGAGAATCCAAGGCTAGCTTAAGCACCGGTGGAGTGAC
793	GTTACCCAGGTCGATTTCAG

794	AAATCGTCCGCTCTATGCAG
814	TCTCATGACCAAAATCCCTTAACGTGAGTTTTCGTTCCACAATGAGTAACAGCTGTAGTTTATCAAAAAGAGTGTTGACTTGTGAGCGGATAACAATG
815	ATGCGGGCTTTGTGCGTTTTAGGGCGTCGGAGATGCTCGAGGAAG
816	CTAAGAAACCAATAAGGCTAAGCCCGGGTGCATGACAGGAAGTTCCTATACTTTCTAG
817	GTACGTACTATCAACAGGTTC
818	CGTTTTTTATTGGTGAGAATCCAAGTAGGGATAACAGGGTAATATAGGTCTGCGAAGTTCCTATACTTTCTAG
819	CCTAAGAAACCAATAAGGCTAAGCCCTATAAACGCAGAAAGGCCCAC
820	TATAGGAACTTCCTGTCATGCACCCGGGCAATACGCAAACCGCCTCTC
874	GCGGGGAGAGGCGGTTTGCGTATTGCCCGCTAGCCCTTAAGGTATACTTTCCGCTGC
875	AAGTATAGGAACTTCCTGTCATGCACCCGGGAGCTTATCGGCCAGCCTCGC
876	CCCTTAACGTGAGTTTTCGTTCCACTAGGGATAACAGGGTAATCGGCTTACCATCCAGCGCCAC
877	ACAAGTCAACACTCTTTTTGATAAACTCTATCGTGCGGTGGTTGAAC
1002	TTGGTGAGAATCCAAGTAGGGATAATTGCGGCCGCAGCTGGCACGACAGGTTTGCCG
1004	AACTTCGCAGACCTATATTACCCTGATGCGGCCGCGACTATGCGGCATCAGAGC
1005	AACTTCGCAGACCTATATTACCCTGTTGCGGCCGCGTCACAGCTTGTCTGTAAGCGGATGCC
1029	CGGGCGTTTTTTATTGGTGAGAATCCAAGTAGGGATAATTTCCTGCACTCTGTGGTCTCA
1030	CGGGCGTTTTTTATTGGTGAGAATCCAAGTAGGGATAATTCCTGCACTCTGTGAAGACAA
1031	TAGAAAGTATAGGAACTTCGCAGACCTATATTACCCTGTTGCCCGGCCACTTCGTGTCCC
1099	CACATTTCCCCGAAAAGTGC
1100	GCACTTTTCGGGGAAATGTGCGCGGAACCCCTATTTGTTTATTTTTC
1132	GAATAGGAACTTCCTCGAGCATCTCCGACGGCGCGCCAGCTGGCACGACAGGTTTG
1133	GAAAAATAAACAAATAGGGGTTCCGCGGGCGCGCCCTATGCGGCATCAGAGCAGATTG
1148	GAACTTCCTCGAGCATCTCCGACGGCGCGCCGATATACCCAAGCTAGACCG
1149	GAAAAATAAACAAATAGGGGTTCCGCGGGCGCGCCCTAGCAAGGATGATCAGAGAC
1152	GAACTTCCTCGAGCATCTCCGACGGCGCGCCGATTCGCATTATTGGCGGATTG
1153	GAAAAATAAACAAATAGGGGTTCCGCGGGCGCGCCCAATCTATCAGGGATAATCC
1156	GAACTTCCTCGAGCATCTCCGACGGCGCGCCTTGCGATAAGCAGTAAAAAACCAC
1157	GAAAAATAAACAAATAGGGGTTCCGCGGGCGCGCCCGCTCACAGACGGAACGATG
1160	GAACTTCCTCGAGCATCTCCGACGGCGCGCCCAGCTCAAGGGGATGAGCAC
1161	GAAAAATAAACAAATAGGGGTTCCGCGGGCGCGCCCGGTTGTTCGACTCTTCAGTG
1162	GAACTTCCTCGAGCATCTCCGACGGCGCGCCGAGCATGCTTCCACACACTC
1163	GAAAAATAAACAAATAGGGGTTCCGCGGGCGCGCCTACGGAAGAATGATCAAGAGTG
1168	GAACTTCCTCGAGCATCTCCGACGGCGCGCCAAGTCGCGTTATGGTAGCAC
1169	GAAAAATAAACAAATAGGGGTTCCGCGGGCGCGCCTCGCCCATAGAGACGGAAAG
1222	CTGGTAGAGATTCGCTATGG
1223	GAGAACAAACCATAGCGAATC
1411	CTTCCTCGAGCATCTCCGACGGCGCGCCCTACGAAAAATACTGGCAGC
1412	AAACAAATAGGGGTTCCGCGGGCGCGCCCTAACCATCAAGGTTGTGTTGTAAATTG
1435	CCTCGAGCATCTCCGACGGCGCGCCTGCAGCTCTGGCCCGTGTCT
1487	GAGCATCTCCGACGGCGCGCCCATGGAGCGGCGTAACCGTC
1488	GGCCAGAGCTGCAGGCGCGCCCGTCGACAGCGACACACTTG
1489	GAGCATCTCCGACGGCGCGCCCCGCCAATGATGATGACGTC
1490	GGCCAGAGCTGCAGGCGCGCCCTGGGATCGTGGGTTAATTTAC
1628	TGGTTTAAATACACCGCCAGCCATGAAAGATGAGGCTGATCAGTTTGTCGTCTCACCGAC
1629	GGGTGGCGCTAACGCTTGATCAATGATCCCTGCCCAGTTAGTCGGTGAGACGACAAACTG
1630	CTTTGCAAATAGTCCTCTTCCAACAATAATAATGTCAGATCCTGTAGACACCACATCATC
1631	TGACAAGGGAGACGCATTGGGTCAACAGTATAGAACCGTGGATGATGTGGTGTCTACAGG

1632	CCCGCAGAGTACTGCAATTTGACTGTATTACCAATGTCAGCAAATTTTCTGTCCTCGAAG
1633	AGCCGCTAAAGGCATTATCCGCCAAGTACAATTTTTTACTCTTCGAGGACAGAAAATTTG
1634	GAAAGTATAGGAACTTCAGAGCGCTTTTGAAAACCAAAAGCGCTCTGATGACGCACTTTC
1635	TTTAGTAGCTCGTTACAGTCCGGTGCGTTTTTGGTTTTTTGAAAGTGCGTCATCAGAGCG
1636	TAACAATTTCACACATACTAGAGAAAGAGGAGAAATACTAGATGGTGAGCAAGGGCGAGG
1637	CTGAGCCTTTCGTTTTATTTGATGCCTGGCTCTAGTATTACTTGTACAGCTCGTCCATGC
1638	ACTGACCAATGCCTCGCTGCACTGGAACATTTTGGCAAGCGCTTTTTGGTCCTCCGCTTC
1639	TGAGAAAGGTAAAGAGATGCAAGCCAAGCTCGATAGTGGTGAAGCGGAGGACCAAAAAGC
1640	TGCGTTCAGTTCAGAAGTTTTAATTGCCATAAGTTACTTACCCCTGATTCAGTGATGACC
1641	AAAAGTGCTTTAGAGCTAGAGCGCGTACTTCATTCACATTGGTCATCACTGAATCAGGGG

Table S4: Primers and templates for the construction of different *Vibrio* replicons

Replicon	gDNA as *oriII* template[a)]	Oligonucleotides
synVituII(amp)	*V. tubiashi*	1166+1167
synVifII(amp)	*V. furnissi*	1156+1157
synViniII(amp)	*V. nigripulchritudo*	1160+1161
synVicoII(amp)	*V. coralliilyticus*	1152+1153
synVipaII(amp)	*V. parahaemolyticus*	1162+1163
synVivuII(amp)	*V. vulnificus*	1168+1169
synViaII(amp)	*V. anguillarum*	1411+1223; 1222+1412

[a)]Strain designations are given in table S1.

Table S5: Relative origin copy numbers derived from CGH experiments[a)]

synVicII-	oriC / ter ratio	oriII / oriC	oriII/ter
candidate 3	3,6	2,6	9,5
	2,9	2,8	8,0
candidate 4	3,4	1,0	3,5
	3,5	1,0	3,6
2.0	3,2	0,7	2,3
	3,9	0,5	2,2

[a)]Numbers are calculated for experiments shown in figures 2, 5 and S2 as indicated.

Supplementary figures S1-S4

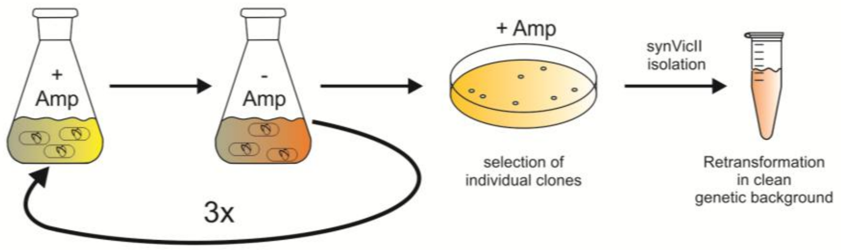

Figure S1 Work-flow scheme for the identification of stabilized synVicII versions. *E. coli* strain SMS18 carrying synVicII-1.3 was grown in LB medium with ampicillin and then shifted to medium without antibiotic (see Material and Methods for details). The process was repeated for three times before cells were plated onto LB agar with ampicillin. synVicII was isolated from these candidate clones and retransformed into *E. coli* MG1655.

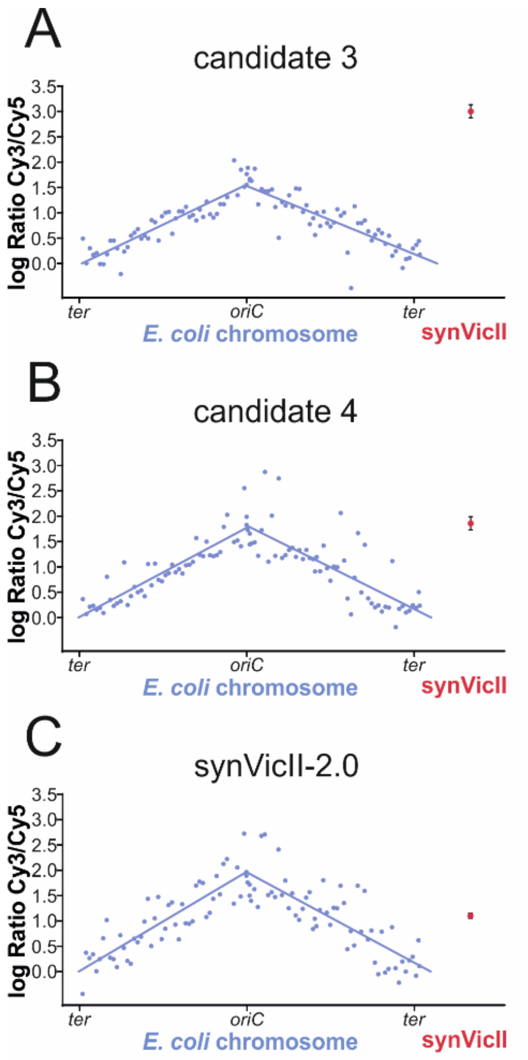

Figure S2 Biological replicates of CGH analyses of *E. coli* strains harboring different versions of synVicII. DNA of exponentially grown cells was hybridized against DNA from rifampicin-runout cells of strain FSK18 (see Materials and Methods for details). Respective logarithmic values of ratios are plotted against their genomic position. Blue dots represent the 104 probes for the *E. coli* chromosome, blue lines the linear curve fitted to chromosome arms. The red dot represents the mean of the three probes of synVicII with the corresponding standard deviation. **A** Strain SMS81 harboring synVicII candidate 3 as in fig. 2E. **B** Strain SMS79 harboring synVicII candidate 4 as in fig. 2F. **C** Strain DS292 harboring synVicII-2.111 as in fig. 5C.

rctB	ACTCTATCGCGTTATACCT**A**TCCAGAAGCGCTAGAACGC
oriII	CCACTAAGTTACGGTGAAT~~G~~CCATTCTGATTGAAATGAT
term$_{gfp}$	TTTGTTCAGAACGCTCGGT**G**GCCGCCGGGCGTTTTTTAT

Figure S3 Point mutations identified by sequencing stabilized synVicII candidates 3, 4 or 5. Respective point mutations are indicated in red. In *rctB* of candidate 3 a cytosine was substituted by adenosine leading to an exchange of serine to tyrosine at codon 555. In *oriII* of candidate 4 and 5, a guanosine was deleted. In the *gfp* terminator of candidate 4, a thymidine was exchanged by guanosine.

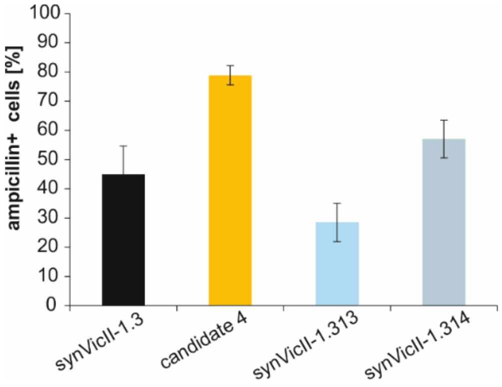

Figure S4 Stability of synVicII versions carrying one of the point mutations found in evolution candidate 4. Given numbers are mean values of ampicillin resistant cells after 6 h of cultivation without antibiotic selection from three biological replicates. Values for synVicII-1.3 and candidate 4 are taken from fig. 2D. synVicII-1.313 carries a mutation in the replication origin and synVicII-1.314 in the transcriptional terminator of the *gfp* gene.

Supplementary references

Blattner, F.R., Plunkett, G., 3rd, Bloch, C.A., Perna, N.T., Burland, V., Riley, M., Collado-Vides, J., Glasner, J.D., Rode, C.K., Mayhew, G.F.*, et al.* (1997). The complete genome sequence of *Escherichia coli* K-12. Science (New York, N.Y.) *277*, 1453-1462.

Heidelberg, J.F., Eisen, J.A., Nelson, W.C., Clayton, R.A., Gwinn, M.L., Dodson, R.J., Haft, D.H., Hickey, E.K., Peterson, J.D., Umayam, L.*, et al.* (2000). DNA sequence of both chromosomes of the cholera pathogen *Vibrio cholerae*. Nature *406*, 477-483.

House, B.L., Mortimer, M.W., and Kahn, M.L. (2004). New recombination methods for *Sinorhizobium meliloti* genetics. Applied and environmental microbiology *70*, 2806-2815.

James, P., Halladay, J., and Craig, E.A. (1996). Genomic libraries and a host strain designed for highly efficient two-hybrid selection in yeast. Genetics *144*, 1425-1436.
Kouprina, N., Annab, L., Graves, J., Afshari, C., Barrett, J.C., Resnick, M.A., and Larionov, V. (1998). Functional copies of a human gene can be directly isolated by transformation-associated recombination cloning with a small 3' end target sequence. Proceedings of the National Academy of Sciences of the United States of America *95*, 4469-4474.
Messerschmidt, S.J., Kemter, F.S., Schindler, D., and Waldminghaus, T. (2015). Synthetic secondary chromosomes in *Escherichia coli* based on the replication origin of chromosome II in *Vibrio cholerae*. Biotechnology journal *10*, 302-314.
Miller, V.L., and Mekalanos, J.J. (1988). A novel suicide vector and its use in construction of insertion mutations: osmoregulation of outer membrane proteins and virulence determinants in *Vibrio cholerae* requires toxR. Journal of bacteriology *170*, 2575-2583.
Schindler, D., Milbredt, S., Sperlea, T., and Waldminghaus, T. (2016). Design and Assembly of DNA Sequence Libraries for Chromosomal Insertion in Bacteria Based on a Set of Modified MoClo Vectors. ACS synthetic biology.
Schlüter, J.P., Czuppon, P., Schauer, O., Pfaffelhuber, P., McIntosh, M., and Becker, A. (2015). Classification of phenotypic subpopulations in isogenic bacterial cultures by triple promoter probing at single cell level. Journal of biotechnology *198*, 3-14.
Weber, E., Engler, C., Gruetzner, R., Werner, S., and Marillonnet, S. (2011). A modular cloning system for standardized assembly of multigene constructs. PloS one *6*, e16765.
